# NUPR1 imparts oncogenic potential in bladder cancer

**DOI:** 10.1002/cam4.5518

**Published:** 2022-12-05

**Authors:** Lifeng Zhang, Shenglin Gao, Xiaokai Shi, Yin Chen, Shuzhang Wei, Yuanyuan Mi, Li Zuo, Chunjian Qi

**Affiliations:** ^1^ Department of Urology The Affiliated Changzhou Second People's Hospital of Nanjing Medical University Changzhou China; ^2^ Department of Urology Affiliated Hospital of Jiangnan University Wuxi China; ^3^ Medical Research Center The Affiliated Changzhou Second People's Hospital of Nanjing Medical University Changzhou China

**Keywords:** bladder cancer, expression, invasion, NUPR1

## Abstract

**Background:**

NUPR1, or p8, is a small chromatin protein that plays a central role in the resistance to treatment and progression of cancer. Nevertheless, the molecular mechanism of NUPR1 in bladder cancer (BLCA) remains unclear.

**Methods:**

We used online databases and immunohistochemistry (IHC) to explore the expression of NUPR1 in BLCA tissues and controls. Lentivirus‐mediated small interfering ribonucleic acid (siRNA) was used to knockdown the expression of NUPR1 in two human BLCA cell lines. We used an in vivo experiment to investigate the effect of NUPR1 knockdown on the growth of BLCA. Moreover, an in silico analysis was conducted to assess the differential expression profile after NUPR1 interference. The CIBERSORT algorithm was utilized to evaluate the effects of tumor‐infiltrating immune cells among BLCA patients.

**Results:**

The expression of NUPR1 in BLCA tissues was significantly higher than in the control. NUPR1 expression was also positively correlated with the stage of BLCA. After lentivirus‐mediated interference, the expression of NUPR1 was significantly down‐regulated in BLCA cell lines. The cell cycle was blocked in G1 phase and the cell proportion of S phase was decreased in both two cell lines. Moreover, in vivo experiment revealed that the tumor growth of BLCA can be delayed by inhibiting the expression of NUPR1. Both in silico analysis and functional experiments revealed that NUPR1 was correlated with epithelial–mesenchymal transition (EMT). We also revealed that macrophages were the most related immune cells associated with the expression of NUPR1 in BLCA.

**Conclusions:**

This study suggests that NUPR1 plays a carcinogenic role in BLCA. NUPR1 lentivirus‐mediated interference could interfere with cycle progression of the BLCA cell, resulting in cell cycle arrest in the G1‐phase. The carcinogenic effect of NUPR1 in BLCA is likely achieved through EMT. NUPR1 is correlated with the M0‐type macrophage markers CD68 and CD11b‐integrin.

## Graphical abstract

We used an in vivo and in vitro experiment to investigate the effect of NUPR1 knockdown on the growth of BLCA. We revealed that NUPR1 lentivirus‐mediated interference could interfere with cycle progression of the BLCA cell, resulting in cell cycle arrest in the G1‐phase. The carcinogenic effect of NUPR1 in BLCA is likely achieved through EMT.

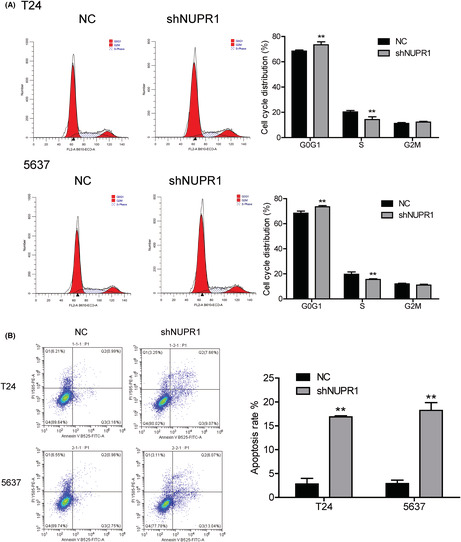

## 1 INTRODUCTION

Bladder cancer (BLCA) is the second most common type of malignant tumor in the genitourinary system. There are 573,278 new diagnoses of BLCA and 212,536 BLCA‐related deaths estimated worldwide.^1^ It is predicted that by 2035, the number of new BLCA patients per year in European countries will increase by 41% from 124,188 in the present to 174,891.^2^ Of any other cancer in the United States, BLCA has the highest cost from diagnosis to death, ranging from $96,000 to $187,000 due to the need for routine lifetime monitoring and treatment.^3^ In China, there is an estimated 91,893 new diagnosed cases and 42,973 BLCA‐related deaths in 2022.^4^ These figures far outstrip the same period in the United States (84,825 new cases and 19,223 estimated deaths). Therefore, strategies for preventing and controlling BLCA are needed to lessen the substantial economic burden on the healthcare system and society. To date, there is still a lack of relatively new and inexpensive molecular urologic tumor markers to predict the occurrence and prognosis of BLCA.^5^

*NUPR1* (also named p8, Com‐1), a gene first discovered in acinar cells of acute pancreatitis, encodes an 82 amino acid monomeric protein (8.8 kDa) with no apparent homology to other proteins.^6^ NUPR1 was originally thought to be a transcription factor due to the presence of a basic helix–loop–helix structure at its C‐terminus, with slight homology to most homeodomains.^6^, ^7^ NUPR1 also has the potential to be phosphorylated by various kinases.^8^ Electrophoretic mobility shift analysis revealed that NUPR1 could bind slightly to deoxyribonucleic acid (DNA), but this binding property can be augmented when phosphorylated by protein kinase A.^9^ Previous reports have shown the abnormal expression of *NUPR1* gene in various benign diseases including diabetic nephropathy, cardiomyocyte hypertrophy, and acute pancreatitis.^10^, ^11^, ^12^ Moreover, various studies have indicated that NUPR1 can participate in various biological functions. NUPR1 is not only an inducer of cell and tumor growth, but also an inhibitor of tumor growth.^13^ However, there are differing opinions on whether NUPR1 has the function of promoting or restricting the growth of tumors.^14^ Additionally, in vitro research results are inconsistent with in vivo research.^13^, ^15^

NUPR1 has the activity of promoting the division of pancreatic cancer cell lines.^16^ The expression of NUPR1 is augmented in most pancreatic cancer patients.^17^ In breast cancer tissues, the mRNA of *NUPR1* was enhanced.^18^ In stratification analysis based on the cancer stage, the expression of NUPR1 was augmented in more advanced breast cancer.^19^ However, no correlation was found between the expression of NUPR1 and different breast cancer subtypes.^19^ Also, NUPR1 expression was attenuated in human prostate cancer cells. The expression of NUPR1 was negatively associated with invasiveness and tumor progression in prostate cancer.^20^ NUPR1 has attracted considerable attention due to its role in apoptosis, DNA repair response, autophagy, and cell cycle regulation.^36^, ^39^ NUPR1 can act as a stress‐induced transcription factor and modulate the stress response to promote the progression of various malignancies.^13^, ^17^ Targeting the NUPR1 with a strong inhibitor has been reported to treat pancreatic adenocarcinoma and hepatocellular carcinoma.^49^, ^50^ Therefore, NUPR1 is promised to be used as a new therapeutic target for treating another carcinoma. However, the prognostic value of NUPR1 in BLCA has not been fully elucidated. Consequently, in the current study, we evaluated the expression of NUPR1 in clinical BLCA tissue. We also conducted a functional experiment to assess the role of NUPR1 in BLCA. Furthermore, an in silico analysis were also used to explore the relative regulatory molecules and signaling pathways associated with NUPR1 in BLCA.

## 2 MATERIALS AND METHODS

### 2.1 Study population

BLCA and para‐cancerous control tissue samples were collected from 80 patients who received transurethral resection of bladder tumor (TURBT) or laparoscopic radical cystectomy (LRC) between January 2015 and December 2020. For patients who underwent LRC, we obtained pairs of tumors and normal bladder tissue adjacent to cancer. However, we did not acquire para‐cancer normal bladder tissue from the patients who received TURBT. The reason is that we did not have the ethical approval to take these specimens. We invited professional pathologists to participate in the process of specimen selection to avoid the interference caused by non‐cancer tissues. The clinicopathologic features including age, body mass index (BMI), gender, tumor stage, and recurrence were shown in Table 1. The diagnosis was according to the pathological examination of the pathology department of Changzhou No.2 People's Hospital. None of these patients received chemotherapy or radiotherapy before surgery. Samples were collected after obtaining the informed consent of the patients. The experiment was approved by the Ethics Committee of Changzhou No.2 People's Hospital (Approval number: [2020]KY223‐01). All the patients have provided the written informed consent. Experiments containing human participants adhered to the Declaration of Helsinki.

**TABLE 1:** Relationship between the expression of NUPR1 and clinicopathologic features of BLCA patients.

Clinicopathological variable	Number	%	NUPR1 expression		
			Low (%)	High (%)	*p* value
Age (years)					
<60	14	17.5	9 (18.74)	5 (15.63)	0.286
61–70	52	65.0	28 (58.33)	24 (74.99)	
>80	14	17.5	11 (22.92)	3 (9.38)	
BMI					
<20	17	21.25	10 (20.83)	7 (21.87)	0.937
20–30	59	73.75	36 (75.00)	23 (71.88)	
>30	4	5.0	2 (4.17)	2 (6.25)	
Gender					
Male	69	86.25	39 (81.25)	30 (93.75)	0.208
Female	11	13.75	9 (18.75)	2 (6.25)	
Stage					
T1	45	56.25	30 (62.50)	15 (46.87)	0.168
≥T2	35	43.75	18 (37.50)	17 (53.13)	
Grade					
Low	46	57.50	35 (72.92)	11 (34.38)	<0.05
High	34	42.50	13 (27.08)	21 (65.62)	
Recurrence					
Yes	15	18.75	5 (10.42)	10 (31.25)	<0.05
No	65	81.25	43 (89.58)	22 (68.75)	

### 2.2 Immunohistochemical analysis

Tissue samples were analyzed by immunohistochemistry (IHC) analysis. The slices were dewaxed with xylene and dehydrated with graded alcohol, and the endogenous peroxidase activity was blocked for 10 min in methanol with 0.5% hydrogen peroxide. Phosphate‐buffered saline (PBS) containing 10% normal goat serum was used to block non‐specific binding at 20°C for 1 h. Then, a rabbit anti‐NUPR1 antibody (1:100, Proteintech) was used to interact with the slides in a moist chamber overnight. Subsequently, the sections were detected with a horseradish peroxidase ligated polymer combined with the corresponding secondary antibody for 10 min. The slices were incubated with 0.1% diaminobenzidine (Sigma) in PBS containing 0.05% hydrogen peroxide at 20°C for 5 min to form a brown precipitate, indicating precipitate peroxidase activity. The staining intensity score ranged from 0 to 3, which represented the staining intensity from shallow to deep, respectively.

### 2.3 Cell culture

The human BLCA cell lines, T24 and 5637, were purchased from the Chinese Academy of Sciences (Shanghai, China). T24 cell lines were derived from a female patient with grade III BLCA. 5637 cell lines were from a grade II BLCA male patient. The cells were prepared in Roswell Park Memorial Institute‐1640 medium (Gibco), including 10% fetal calf serum, 100 U/ml penicillin and streptomycin in a 5% carbon dioxide (CO2) incubator at 37°C. The culture media of these two cell lines were changed every 3 days. The cells were harvested when they reached about 80% confluence.

### 2.4 RT‐qPCR of endogenous NUPR1 and transfection of lentivirus

We used a total RNA extractor kit (Pufei Corp.) to extract RNA from the two BLCA cell lines. Then we applied Moloney Murine Leukemia Virus reverse transcriptase (Promega) with random primers to reverse transcribe RNA into cDNA. The primer sequences of *NUPR1* were 5’‐ATAGCCTGGCCCATTCCTAC‐3′ (forward) and 5’‐GCAGCAGCTTCTCTCTTGGT‐3′ (reverse). The primer sequences of Glyceraldehyde 3‐phosphate dehydrogenase (*GAPDH*), an internal control, were 5’‐TGACTTCAACAGCGACACCCA‐3′ (forward) and CACCCTGTTGCTGTAGCCAAA (reverse). Real‐time quantitative‐polymerase chain reaction (RT‐qPCR) was conducted using a real‐time polymerase chain reaction (PCR) detection kit (Takara). The RT‐qPCR method involved heating at 95°C for 30 s, followed by denaturation at 95°C for 5 s and then 59°C for 30 s for 40 cycles. The relative expression of NUPR1/GAPDH mRNA was investigated using the 2^−∆∆Ct^ method. Lentivirus‐mediated small interfering RNA (siRNA) was used to knockdown the expression of NUPR1 in two human BLCA cell lines. The RNA interference sequence of NUPR1 was designed: CCAAGCTGCAGAATTCAGA. The negative control (NC) was a non‐silencing small‐interfering RNA (siRNA) sequence that did not target any mammalian genes. The efficiency of *NUPR1* viral interference in BLCA cell lines (T24 and 5637) was detected by RT‐qPCR.

### 2.5 Cell cycle and apoptosis assay

When the density of cultured cells reached 85%, the lentivirus was added. Then these cells were rinsed once with PBS, digested with trypsin, and counted with a blood cell counter to ensure enough cells per well. The cells were moved to a 15 ml test tube and centrifuged at 1000 rpm. The supernatant was removed, and the cell particles were rinsed twice with PBS. The flow cytometry (FCM) analysis was conducted according to protocols of Becton & Dickinson (BD) Biosciences with the flow rate of 100–150 cells/s. NUPR1‐siRNA and the control cell cultures were digested with trypsin and suspended in the logarithmic phase for the apoptosis assay. The phycoerythrin (PE) Annexin V apoptosis kit (BD Biosciences) was used to evaluate cell apoptosis. The concentration of cells was 1 × 10^6^ ml, diluted with 1 × staining buffer. To the binding buffer, 5 μl of Annexin V‐PE and 7‐aminoactinomycin D were added, and staining was conducted at room temperature in the dark for 15 min.

### 2.6 Cell migration and invasion

The Matrigel matrix glue, frozen in the refrigerator at −20°C was placed overnight at 4°C and diluted to a 1:5 working liquid. The upper ventricular surface of the bottom membrane of the Transwell chamber was added and placed in a 37°C incubator for 30 min to gelatinize the Matrigel. The cells were cultured in a serum‐free medium for 12 h and rinsed three times with PBS. The 1 × 10^5^/ml cell suspension was made from serum‐free medium for the subsequent experiment. Then, the 100 μl cell suspension and 200 μl serum‐free medium were added to the upper chamber of the Transwell culture plate. The 600 μl culture medium containing 5% fetal bovine serum (FBS) was added into the lower chamber and incubated at 37°C with 5% CO_2_ for 24 h. The chamber was taken out, and cells were removed from the inside of the chamber with a cotton swab. Then they were fixed for 5 min with 95% alcohol and stained with 4 g/L crystal violet solution. Three visual fields were randomly selected and counted using the high‐power mirror, and the average was calculated.

### 2.7 Western blotting

We used a protein cleavage buffer combined with a 1% protease inhibitor cocktail to extract the total protein. The primary antibodies utilized were: anti‐NUPR1 (Proteintech group), anti‐Vimentin (Abcam), anti‐E Cadherin (Abcam), and anti‐GAPDH (Abcam). Polyacrylamide gel electrophoresis was used to separate the proteins. Samples were then transferred to a polyvinylidene fluoride (PVDF) membrane. Subsequently, PVDF membranes were sealed using 5% skim milk. The membranes and the first antibody in the sealing buffer were incubated overnight at 4°C. Then the PVDF membranes were rinsed with 1 × tris‐buffered saline and incubated with a secondary antibody at room temperature for 1 h. Chemiluminescence reagent (Millipore) and ImageJ software were used to investigate and quantify the bands. The GAPDH protein was used as an internal control.

### 2.8 Subcutaneous tumorigenesis in nude mice

Nude mice were raised in a sterile feeding room. The male nude mice (aged 4–6 weeks) were randomly divided into groups (experimental and control group) with five mice in each group. T24 cells with low expression of NUPR1 were injected subcutaneously into the experimental group. The infected T24 cells and control cells in the logarithmic growth phase were collected. The 100 μl single cell suspension containing 2 × 10^6^ cells was prepared after rinsing twice with PBS. Then, the cell suspension was extracted for injection. The injection site was between the scapulae of the nude mice. After the tumor was formed, the growth of the tumor was recorded. The tumor size was measured with a Vernier caliper, the volume was calculated, and the growth curve was drawn. After 35 days, the nude mice were killed, and the tumor was removed and weighed. Experiments containing animals were based on the ARRIVE guidelines.

### 2.9 Statistical and in silico analyses of NUPR1

We used R language to evaluate clinical data downloaded from The Cancer Genome Atlas (TCGA) database. The IHC analyses data were measured using Graphpad Prism software (version 7.0). The results were presented as the mean ± standard deviation. Additionally, we adopted a Student's *t*‐test to measure the mean of measurement data. The continuous variables of two or more groups were tested by a one‐way analysis of variance, where *p* < 0.05 was considered statistically significant. All experimental data came from three independent experiments. Moreover, gene set enrichment analysis (GSEA) was employed to explore the possible signaling pathways correlated with the expression of NUPR1. We adopted c2.cp.kegg.v7.1.symbols.gmt as the reference gene set.^21^ The CIBERSORT algorithm was utilized to evaluate the effects of tumor‐infiltrating immune cells (TICs) in BLCA patients.^22^

## 3 RESULTS

### 3.1 Expression of NUPR1 in clinical tissue of BLCA

**FIGURE 1:**
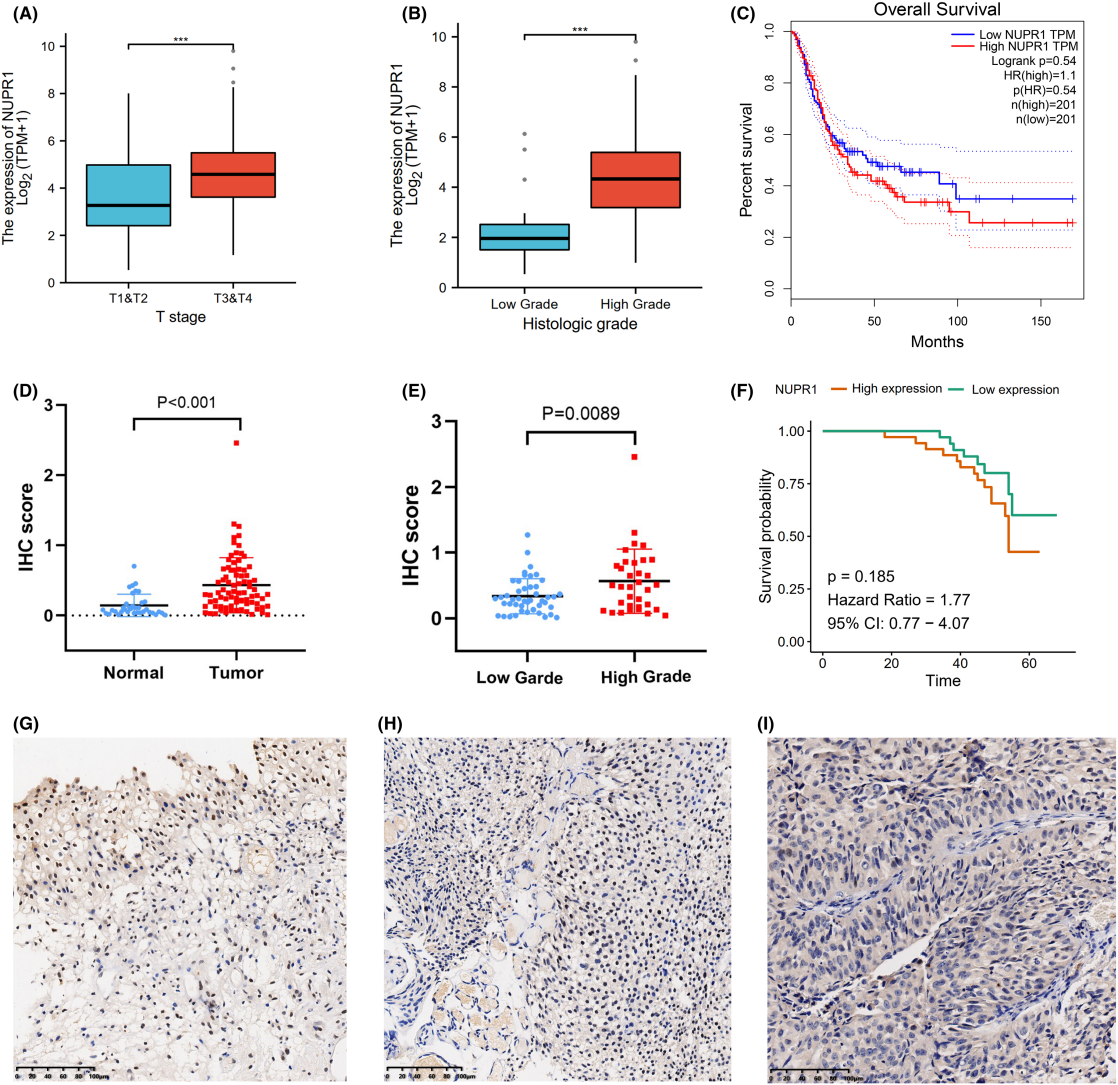
Association between NUPR1 expression and clinical manifestation of bladder cancer (BLCA). (A) Evidence from TCGA database indicated that the expression of NUPR1 in T3 and T4 BLCA was higher than that in T1 and T2 cancer (***p < 0.001). (B) The expression of NUPR1 was positively correlated with the grade of BLCA in TCGA database (***p < 0.001). (C) showed the Kaplan–Meier curves of overall survival time between high and low NUPR1 expression groups in TCGA database. (D) Evidence from BLCA patients enrolled in our centers showed that the expression of NUPR1 in BLCA tissues was significantly higher than that in control (*p* < 0.001). (E) The expression of NUPR1 in high‐grade BLCA was significantly higher than that in low‐grade cancer (*p* < 0.01). The overall survival time between high and low NUPR1 expression groups in cohorts from our hospital was shown in (F). (G): NUPR1 was mainly chromogenic in the nucleus of normal tissues adjacent to cancer. Low expression level of NUPR1 in normal tissues adjacent to cancer. The scale bar is from 0 to 100 μm. (H): Immunohistochemical analysis of NUPR1 in low‐grade BLCA. NUPR1 was stained in both cytoplasm and nucleus of low‐grade BLCA. (I) High expression level of NUPR1 in high‐grade BLCA. The staining intensity of NUPR1 in high‐grade BLCA was significantly stronger than that in low‐grade group.

We used the TCGA database to investigate the relationship between NUPR1 expression and the clinical manifestation of BLCA. The expression of NUPR1 in T3 and T4 BLCA was higher than that in T1 and T2 cancer (Figure 1A, *p* < 0.001). Moreover, the expression of NUPR1 was positively correlated with the grade of BLCA in TCGA database (Figure 1B, *p* < 0.001). Moreover, we investigated the Kaplan–Meier curves of overall survival time between high and low NUPR1 expression groups in TCGA database (Figure 1C). Despite *p* > 0.05, high NUPR1 expression group may be associated with a shorter overall survival time than low NUPR1 expression group. To verify the results from the online database, we conducted an IHC analysis to explore the expression of NUPR1 in tissues from BLCA patients enrolled in our centers. As described in Table 1, the association between the expression of NUPR1 and clinicopathologic features of BLCA patients was assessed. A total of 80 patients were included in our study. The results revealed that the expression level of NUPR1 was attenuated in normal tissues adjacent to cancerous tissues. NUPR1 was mainly chromogenic in the nucleus of normal tissues adjacent to cancer (Figure 1G). Additionally, the expression of NUPR1 in BLCA tissues was significantly higher than that in adjacent normal tissues (Figure 1D, *p* < 0.001). NUPR1 was stained in both cytoplasm and nucleus of low‐grade BLCA (Figure 1H). The expression of NUPR1 in high‐grade BLCA was significantly higher than that in low‐grade cancer (Figure 1E,I, *p* < 0.01). The overall survival time between high and low NUPR1 expression groups in cohorts from our hospital was shown in Figure 1F. The overall survival outcomes of patients enrolled from our centers were consistent with that from TCGA database.

### 3.2 Efficiency of NUPR1 viral interference in BLCA cell lines

**FIGURE 2:**
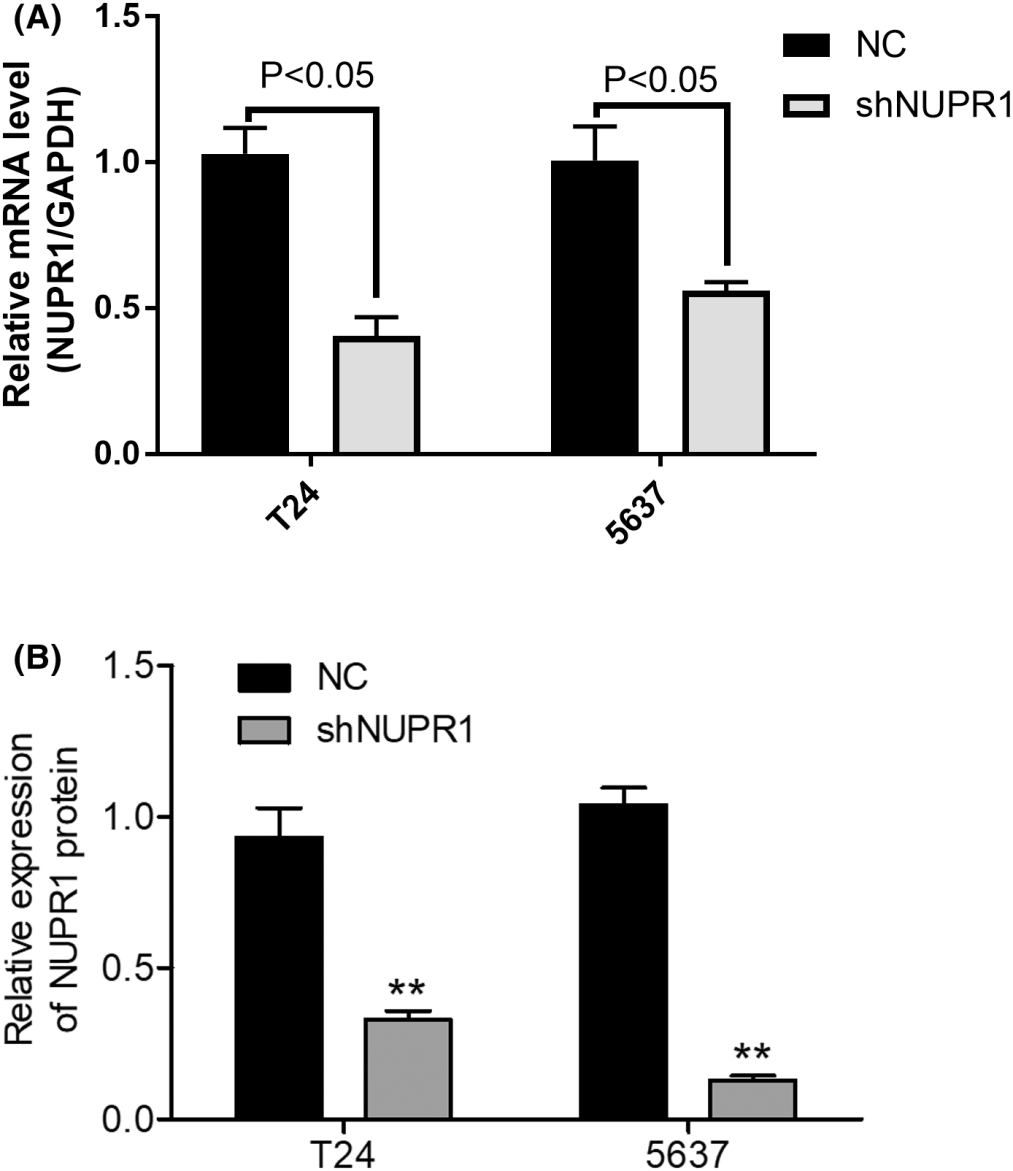
The efficiency of NUPR1 interference virus in BLCA cell lines (T24 and 5637) detected by real‐time (RT) quantitative PCR and Western blotting. RT‐qPCR showed that the expression of NUPR1 was significantly down‐regulated in T24 cell line after interference of virus (*p* < 0.05, A). Similar results were revealed in 5637 cell line (*p* < 0.05). Western blotting analysis revealed that the NUPR1 expression was also attenuated in T24 and 5637 cell lines after interference (*p* < 0.01, B). NC, negative control.

**FIGURE 3:**
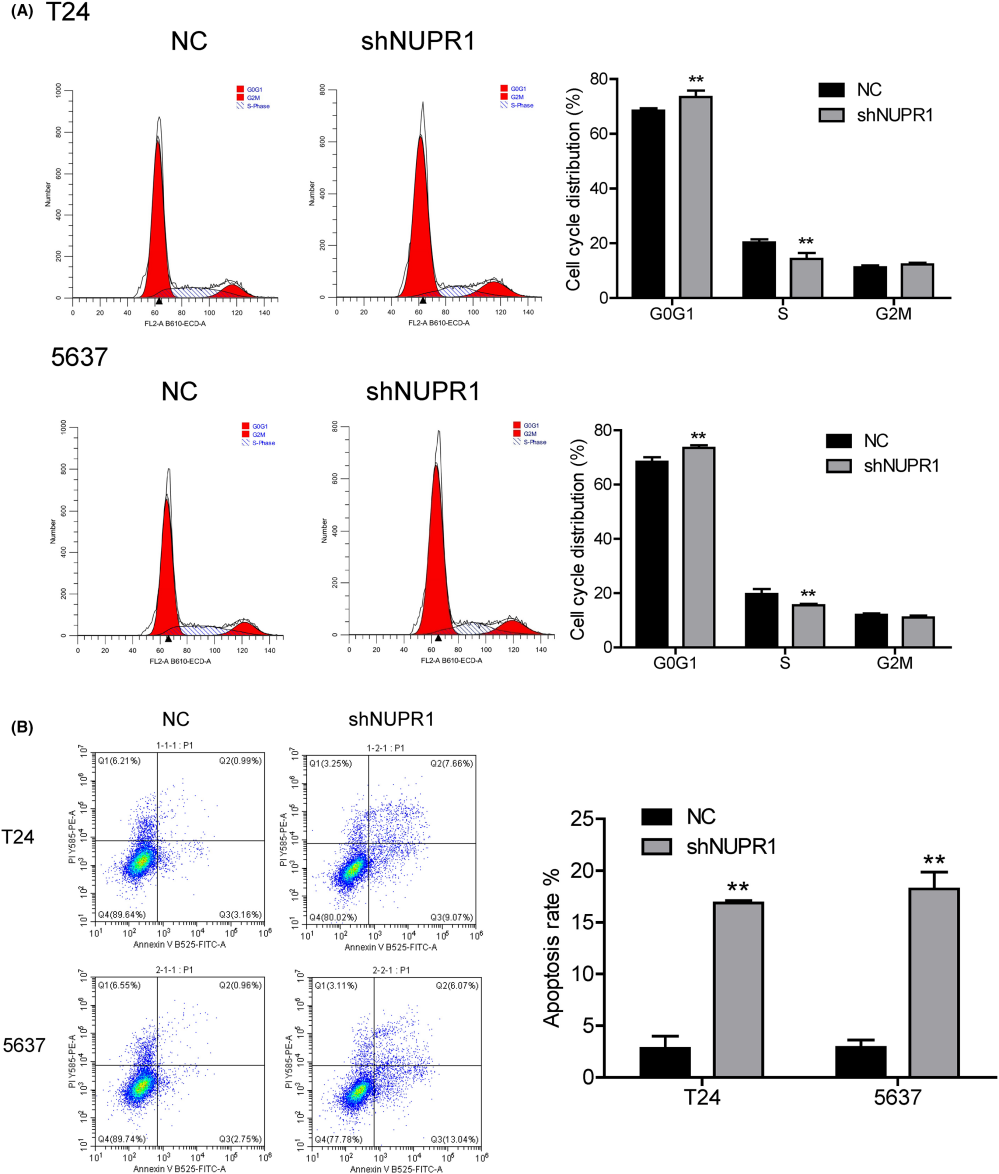
Effects of NUPR1 interference on cell cycle and apoptosis. As compared with control group, G1 phase arrest occurred in both T24 and 5637 cell lines after interference of NUPR1. An obvious down‐regulation of cell population in the S‐phase was revealed (A, **p < 0.01). The apoptosis of T24 and 5637 cells augmented significantly after interference of NUPR1 determined by flow cytometric analysis (B, **p < 0.01). NC, negative control.

The T24 and 5637 cell lines were utilized to evaluate the effect of NUPR1 lentivirus‐mediated interference. Results from the RT‐qPCR revealed that the expression of NUPR1 was significantly down‐regulated in the T24 cell line after the interference effect of lentivirus (Figure 2A). Similar results were also obtained in 5637 cell line (*p* < 0.05). Western blotting analysis revealed that the NUPR1 expression was also attenuated in T24 and 5637 cell lines after interference (*p* < 0.01, Figure 2B). Furthermore, we further investigated the impacts of NUPR1 interference on the cell cycle and apoptosis. As shown in Figure 3A, G1‐phase arrest occurred in both T24 and 5637 cell lines compared to the control group after NUPR1 interference. An apparent down‐regulation of the cell population in the S‐phase was revealed (*p* < 0.05). These findings support that the NUPR1 lentivirus‐mediated interference could interfere with the BLCA cell cycle progression, resulting in cell cycle arrest in the G1‐phase. The apoptosis of T24 and 5637 cells was significantly augmented after NUPR1 interference determined by flow cytometric analysis (**Figure** **3** **B**, *p* < 0.05). This finding demonstrated that NUPR1 knockdown could induce cell apoptosis in BLCA cells.

### 3.3 Cell migration and invasion

**FIGURE 4:**
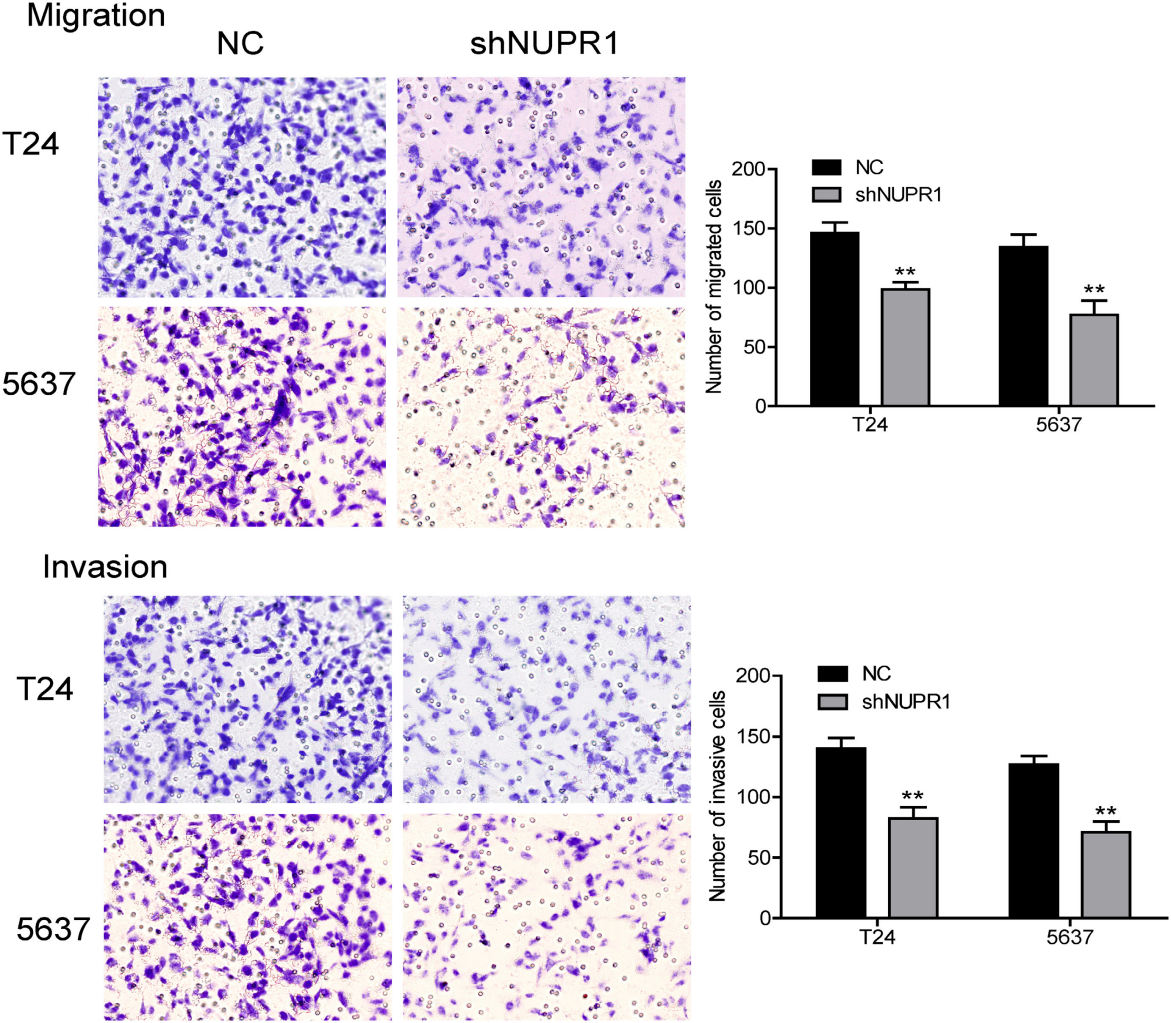
Function of NUPR1 investigated by cell migration and invasion experiment. Transwell experiment showed that the migration abilities of T24 and 5637 cells were attenuated significantly after interference of NUPR1 (**p < 0.01). Similarly, the invasive abilities of these two cell lines were also diminished in NUPR1 interference group, compared with control group (**p < 0.01). NC, negative control.

The Transwell experiment showed that the migration abilities of T24 and 5637 cells were attenuated significantly after NUPR1 interference (*p* < 0.05). Similarly, the invasive abilities of these two cell lines were also diminished in the NUPR1 interference group, compared to those in the control group (Figure 4, *p* < 0.05). These data indicated that NUPR1 could promote the invasion and migration of BLCA cell lines.

### 3.4 Influence of NUPR1 gene knockdown on the growth of BLCA in vivo

**FIGURE 5:**
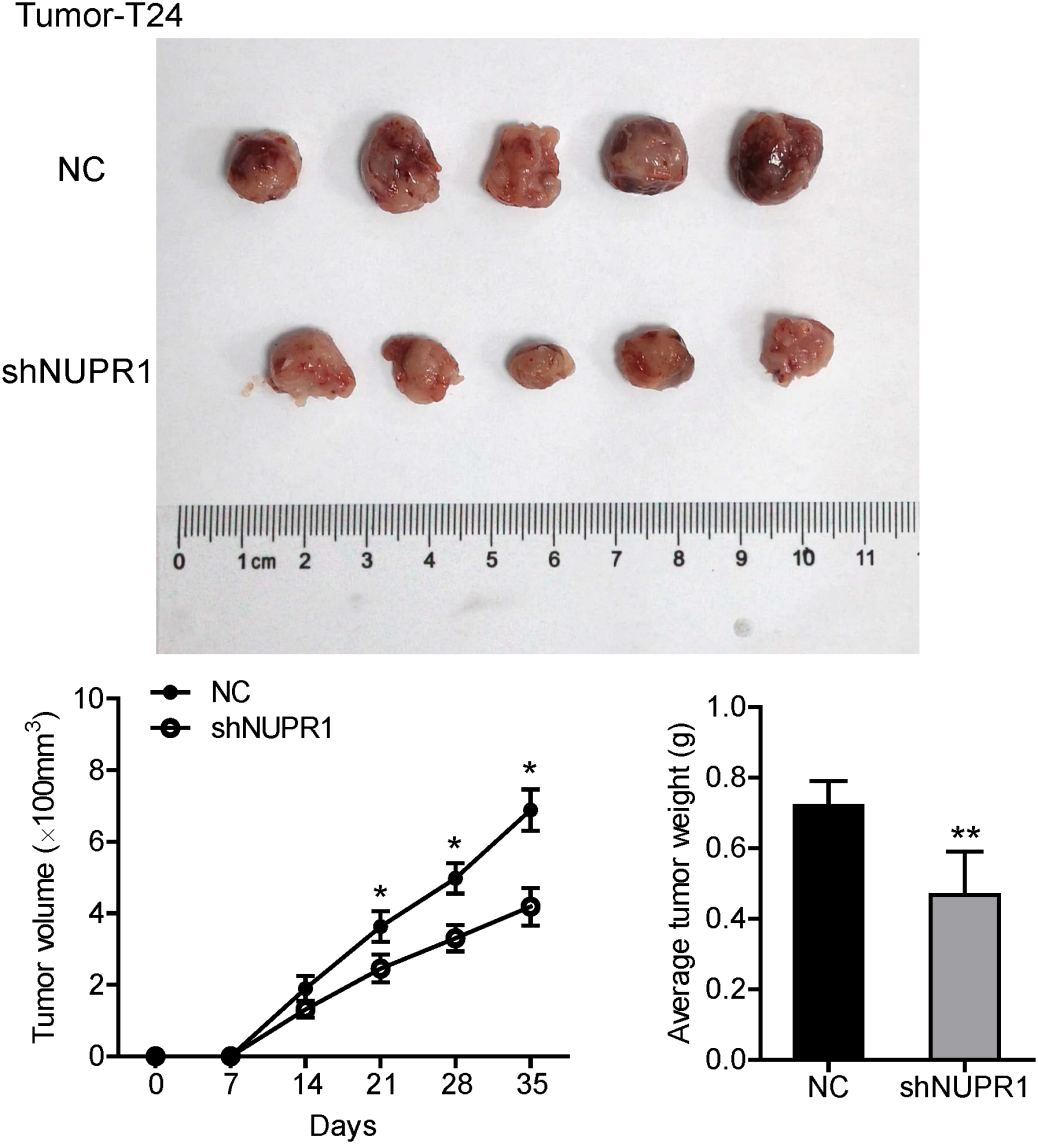
Function of NUPR1 assessed by animal experiment. The subcutaneous tumorigenesis of T24 cells in nude mice was used to verify the function of NUPR1. Compared with the control group, the volume and weight of tumor in shNUPR1 group was attenuated significantly (*p < 0.05, **p < 0.01). We adopted a Student's t‐test to measure the mean of measurement data. NC, negative control.

The subcutaneous tumorigenesis of T24 cells in nude mice was used to verify the function of NUPR1. Compared to the control group, the volume and weight of tumors in the NUPR1 knockdown (KD) group were attenuated significantly (Figure 5, *p* < 0.05). Results from the in vivo experiment also revealed that the tumor growth of BLCA can be delayed by inhibiting the expression of NUPR1.

### 3.5 In silico analyses and identification of NUPR1‐related proteins

**FIGURE 6:**
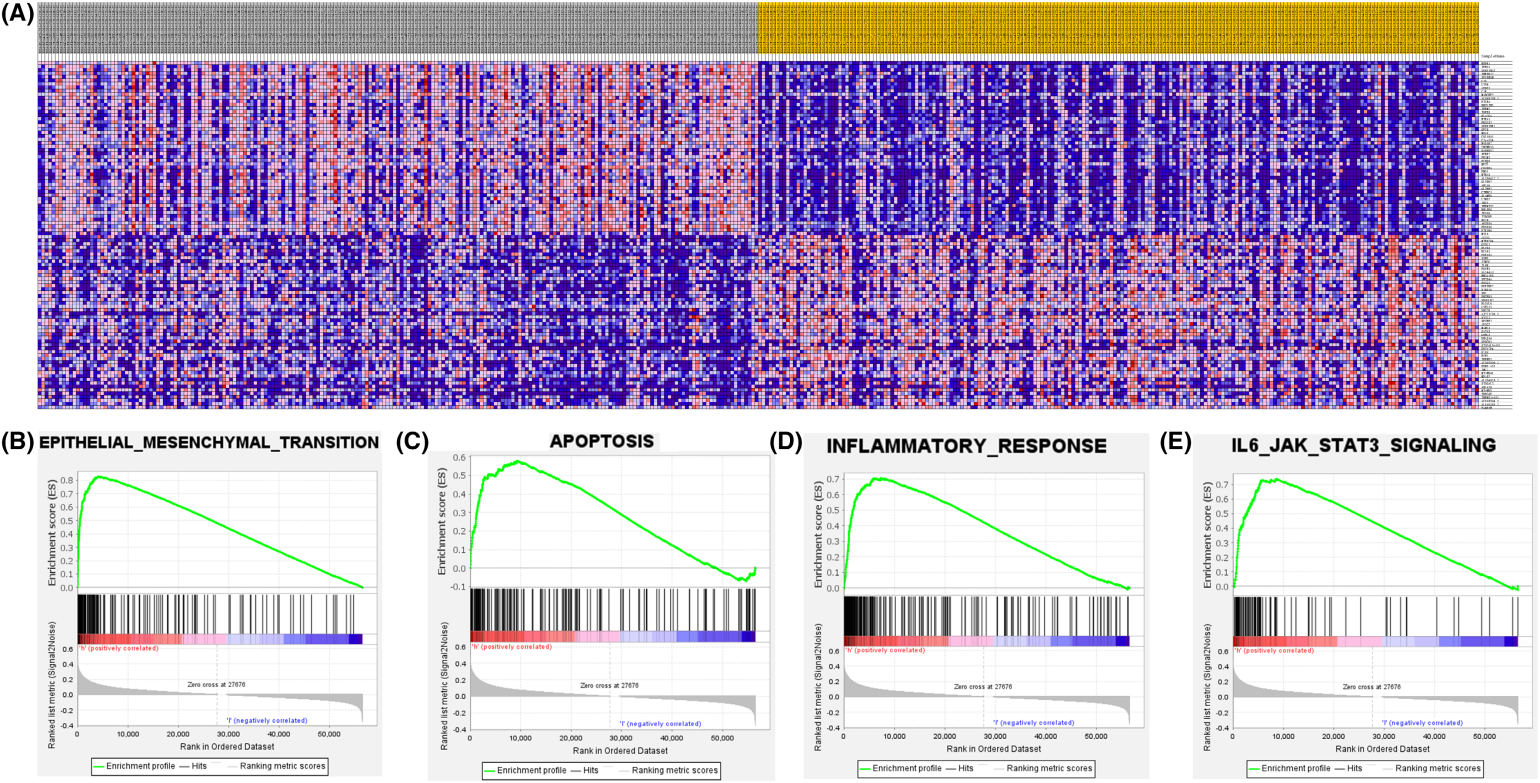
The expression profile of NUPR1 investigated by Gene Set Enrichment Analysis (GSEA). Heat map of GSEA were described in (A). Signaling pathway of EMT (B), apoptosis (C), inflammatory (D), and IL6‐JAK‐STAT3 (E) were correlated with high expression of NUPR1.

**FIGURE 7:**
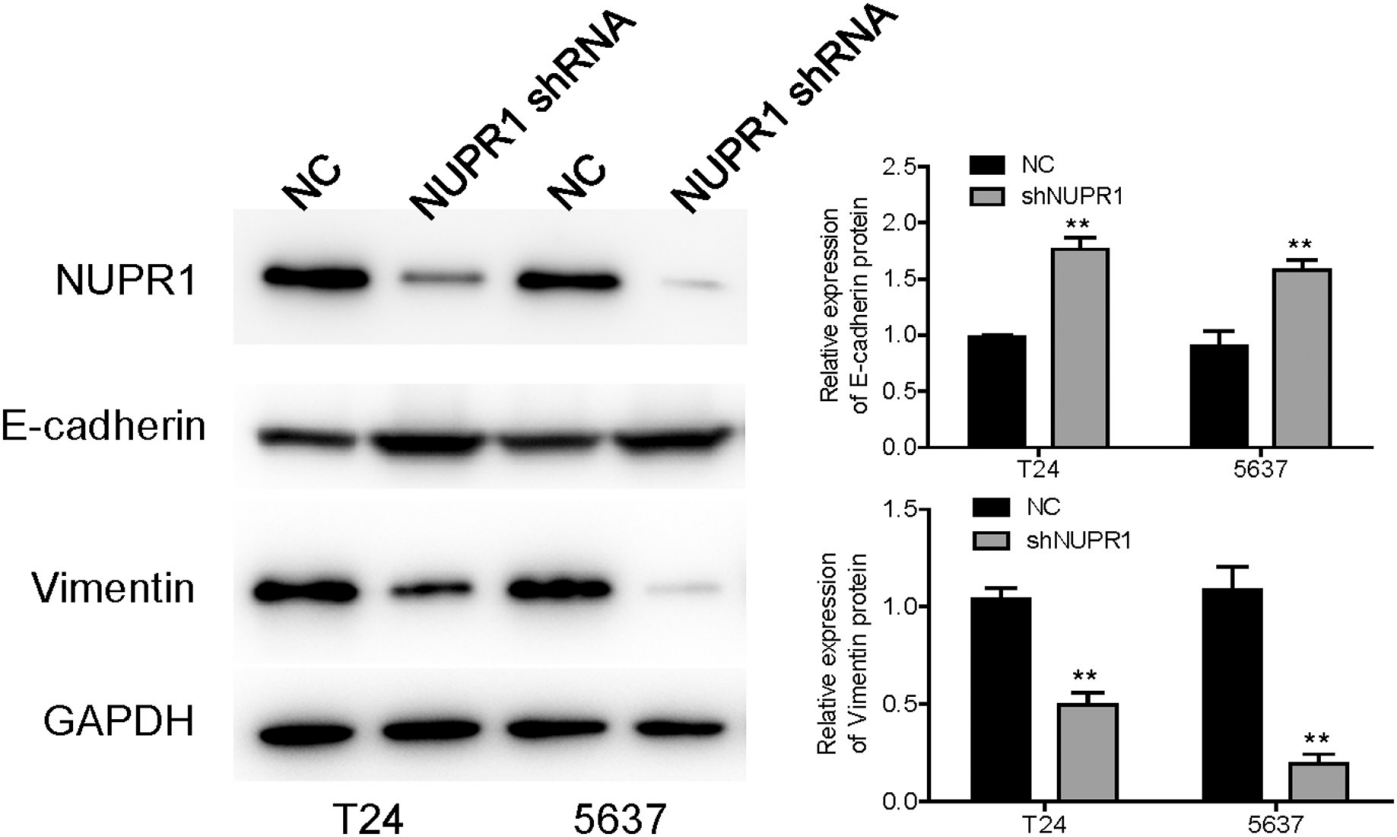
NUPR1 was correlated with EMT. Western blotting analysis showed that E‐cadherin was significantly ameliorated in T24 and 5637 cells after NUPR1 interference (**p < 0.01). Conversely, the expression of Vimentin was significantly down‐regulated in NUPR1 interference group, compared with control group (**p < 0.01). These results showed evidence that NUPR1 could participate in the process of EMT. NC, negative control.

**FIGURE 8:**
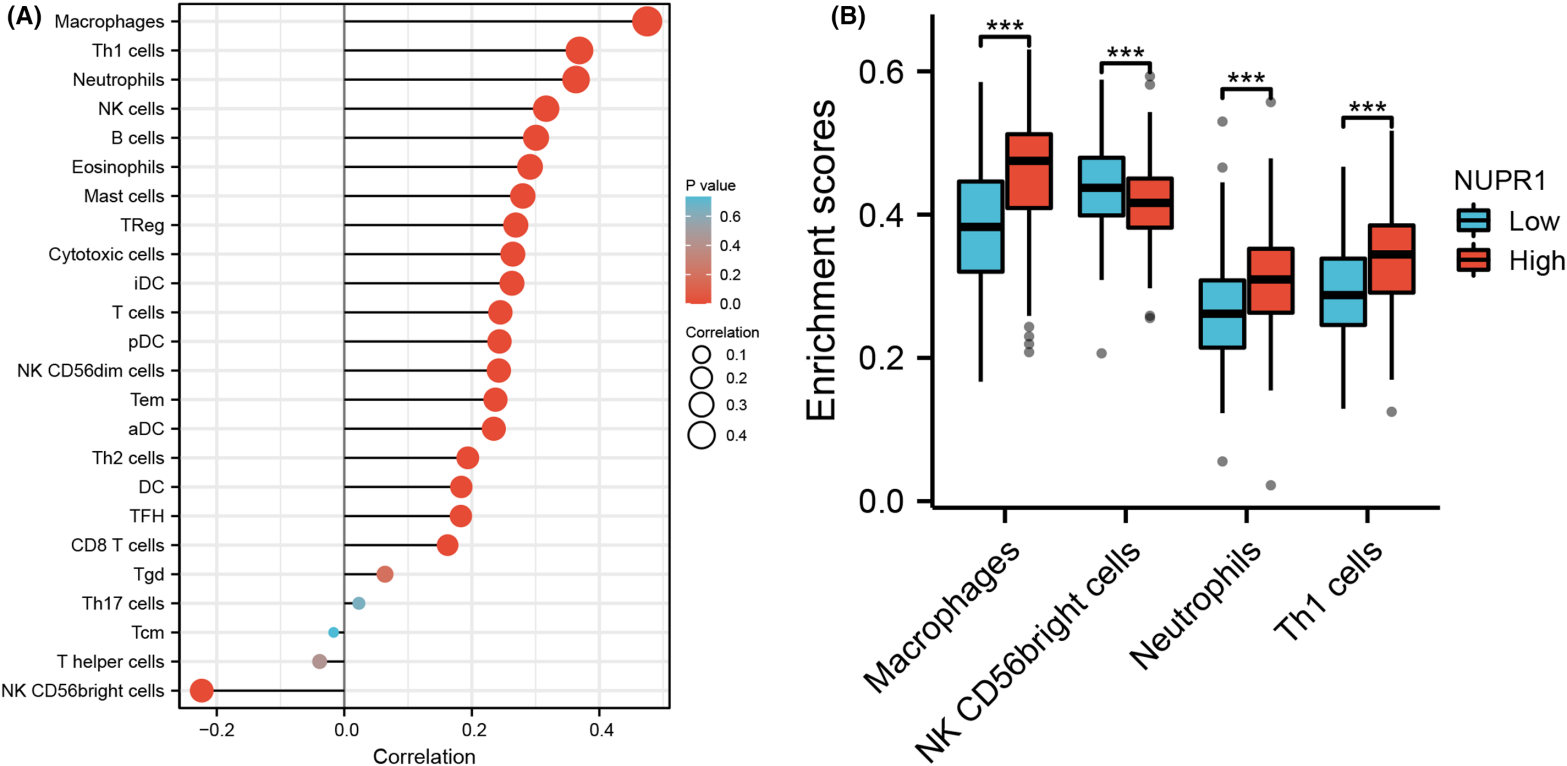
Correlation analysis of NUPR1 and immune cells. The association between NUPR1 and 24 kinds of immune cells was described in Figure A. Figure B listed the immune cells with the most significant difference between the high and low expression groups of NUPR1 in BLCA. macrophages, Th1 cells, neutrophils, and NK CD56 bright cells were the immune cells with the highest correlation with the expression of NUPR1. ***p < 0.001.

**FIGURE 9:**
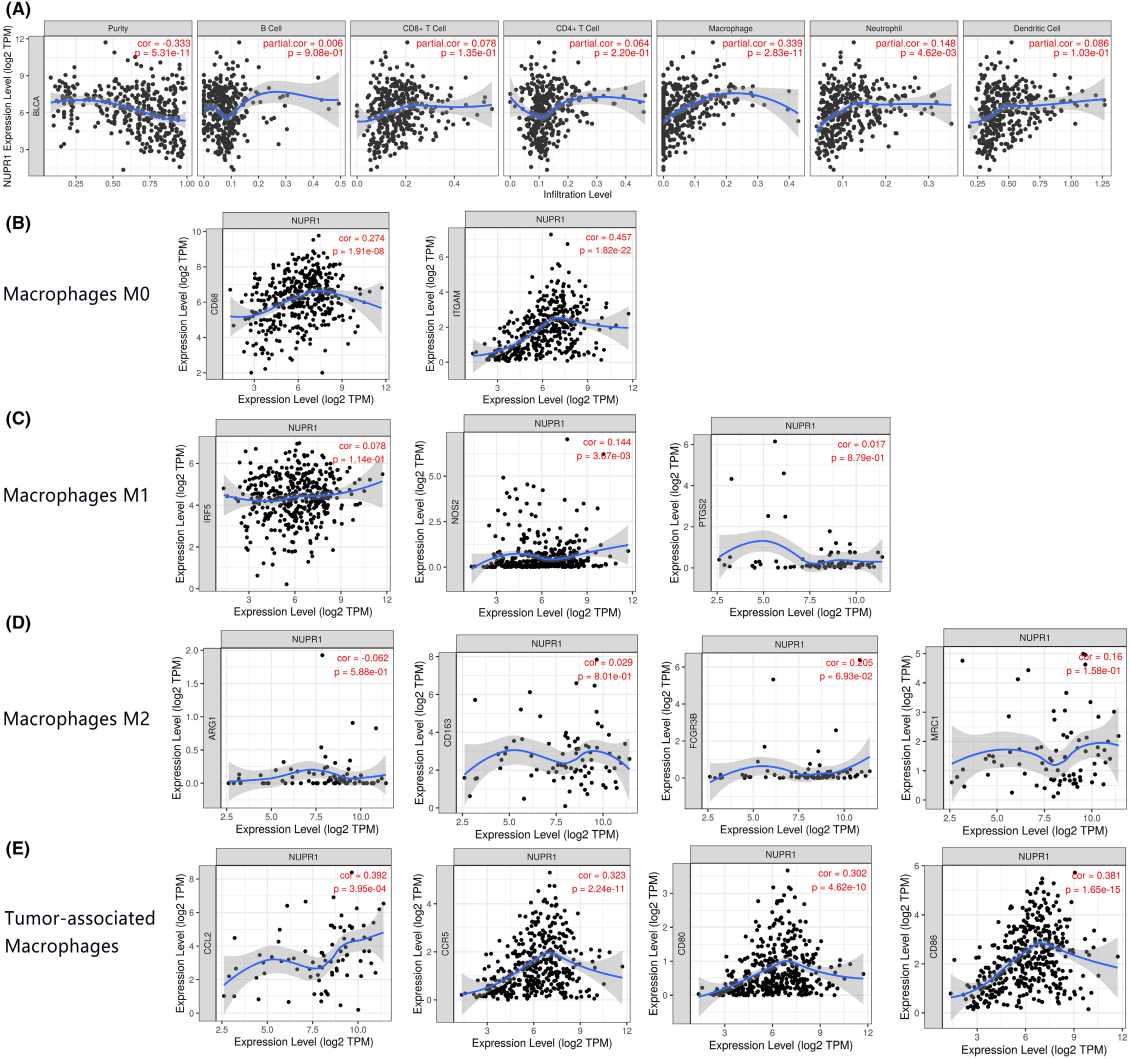
The correlation between the expression of NUPR1 and tumor‐infiltrating immune cells (TICs). Correlation between the expression level of NUPR1 and infiltration level of six kinds of immune cells was shown in (A). The NUPR1 expression was positively correlated with the infiltration level of macrophages (*R* = 0.339, *p* < 0.001) and neutrophils (*R* = 0.148, *p* < 0.004). We further investigated the association between the NUPR1 expression and macrophages cell surface markers. The expression of NUPR1 was highly correlated with M0 type macrophage marker CD68 (*R* = 0.274, *p* < 0.001) and ITGAM (*R* = 0.457, *p* < 0.001, B). There was a weak correlation between the expression of NUPR1 and the M1 macrophage marker NOS2 (*R* = 0.144, *p* < 0.003, C). No significant correlation was revealed between the expression of NUPR1 and the infiltration of M2 macrophages (*p* > 0.05, D). The expression of NUPR1 was highly correlated with tumor‐associated macrophage markers including CCL2 (*R* = 0.392, *p* < 0.001), CCR5 (*R* = 0.323, *p* < 0.001), CD80 (*R* = 0.302, *p* < 0.001) and CD86 (*R* = 0.381, *p* < 0.001) (E).

We utilized GSEA to explore the possible signaling pathways correlated with the expression of NUPR1. Heat maps of GSEA were shown in Figure 6A. The GSEA showed evidence that the signaling pathways including EMT (Figure 6B), apoptosis (Figure 6C), inflammatory (Figure 6D), and interleukin‐6/Janus kinase/signal transducer and activator of transcription 3 (IL6‐JAK‐STAT3), (Figure 6E) were correlated with a high expression of NUPR1. To verify the result of the in silico analysis, we used western blotting (WB) to identify NUPR1‐related proteins, which showed that E‐cadherin was significantly increased in T24 and 5637 cells after NUPR1 interference (Figure 7, *p* < 0.05). Conversely, the expression of Vimentin was significantly down‐regulated in the NUPR1 interference group, compared with that in the control group (*p* < 0.05). These results provided evidence that NUPR1 could participate in the EMT process. Therefore, *NUPR1* may exert its biological function in BLCA by affecting EMT. Moreover, we employed the CIBERSORT algorithm to investigate the association between NUPR1 expression and TICs in BLCA patients. The association between NUPR1 and 24 kinds of immune cells was described in Figure 8A. We also revealed the most related immune cells associated with the expression of NUPR1 in BLCA. macrophages, Th1 cells, neutrophils, and NK CD56 bright cells were the immune cells with the highest correlation with the expression of NUPR1 (**Figure** **8** **B**, *p* < 0.001). The correlations between the expression level of NUPR1 and the infiltration levels of six kinds of immune cells were shown in Figure 9A. NUPR1 expression was positively correlated with the infiltration level of macrophages (*R* = 0.339, *p* < 0.001) and neutrophils (*R* = 0.148, *p* < 0.004). Additionally, we investigated the association between NUPR1 expression and macrophage cell surface markers. The expression of NUPR1 was highly correlated with the M0 type macrophage markers CD68 (*R* = 0.274, *p* < 0.001) and CD11b‐integrin (ITGAM, *R* = 0.457, *p* < 0.001, Figure 9B). There was also a weak correlation between the expression of NUPR1 and the M1 macrophage marker nitric oxide synthase 2 (NOS2, *R* = 0.144, *p* < 0.003, Figure 9C). No significant correlation was revealed between the expression of NUPR1 and the infiltration of M2 macrophages (*p* > 0.05, Figure 9D). The expression of NUPR1 was highly correlated with tumor‐associated macrophage markers including C‐C motif chemokine ligand 2 (CCL2, *R* = 0.392, *p* < 0.001), C‐C chemokine receptor type 5 (CCR5, *R* = 0.323, *p* < 0.001), CD80 (*R* = 0.302, *p* < 0.001), and CD86 (*R* = 0.381, *p* < 0.001) (Figure 9E).

## 4 DISCUSSION

Cancer remains the most significant cause of death and a notable obstacle to extending life expectancy worldwide. Researchers worldwide are actively exploring tumor pathogenesis and predisposing factors, although they have not been fully elucidated.^23^, ^24^, ^25^, ^26^ The extensive invasion of BLCA can greatly impact on the poor prognosis of BLCA patients.^27^ Therefore, the diagnosis and therapy of BLCA might be improved by investigating target molecules.^28^ In the present study, we evaluated the expression of NUPR1 in clinical BLCA tissue. Furthermore, we used lentivirus‐mediated siRNA to knockdown the expression of NUPR1 in two human BLCA cell lines. The in vivo experiment was also conducted to investigate the impact of NUPR1 on the biological behavior of BLCA. We hope to provide a new strategy for the future development of bladder cancer drug therapy by exploring the biological function of NUPR1 in BLCA.

Cancer cells can form a series of mechanisms to cope with stressful environmental conditions such as hypoxia, toxins, or drugs.^29^, ^30^, ^31^ Some stress proteins can be activated by these adaptative mechanisms to promote the survival, proliferation, and progression of cancer cells.^32^, ^33^ Previous studies have demonstrated that the biological function of malignant tumor cells is highly dependent on the activities of stress‐induced proteins.^34^, ^35^ Therefore, it is helpful to clarify the mechanism of tumor drug resistance and explore promising targets in tumor treatment by studying the mechanism of these stress factors. NUPR1 is a crucial stress response transcription factor first identified to be activated in acute pancreatitis.^6^ Subsequent research has shown that NUPR1 can be up‐regulated by various chemical or biological stressors.^8^, ^36^ NUPR1 was involved in several cancer‐related processes, such as apoptosis, autophagy, cell cycle, and metastasis.^37^, ^38^, ^39^, ^40^ Previous studies have explored the dysregulation of NUPR1 in numerous malignant tumors, such as brain tumors, lung cancer, breast cancer, colorectal cancer, and prostate cancer.^41^, ^42^, ^43^ However, the specific mechanism of NUPR1 in malignant tumors is still unclear. Jiang et al. found that NUPR1 was down‐regulated in prostate cancer and that NUPR1 over‐expression can inhibit the growth of tumors.^20^ This finding was unable to be verified by another researcher, who indicated that up‐regulation of NUPR1 can promote cell docetaxel chemoresistance in prostate cancer.^43^

Up to the present, the biological role of NUPR1 in BLCA has not been clarified. Consequently, in the current study, we used an online database to explore the expression of NUPR1 in BLCA. We found that the expression of NUPR1 in high‐grade BLCA was higher than that in low‐grade cancer. Additionally, NUPR1 expression was positively correlated with the stage of BLCA. We also used immunohistochemical analysis to assess NUPR1 expression in BLCA patients enrolled in our centers and the expression of NUPR1 was augmented in BLCA tissues. Our findings agreed with Yu et al., who demonstrated an oncogenic effect of NUPR1 in ovarian cancer.^44^ Moreover, an in silico analysis was conducted to explore the relative regulatory molecules and signaling pathways associated with NUPR1 in BLCA. Our results were consistent with previous studies based on the tissue response of the pancreas, liver, and lung.^45^We further used GSEA to explore the possible signaling pathways correlated with the expression of NUPR1. Signaling pathways, including EMT, apoptosis, inflammatory, and IL6‐JAK‐STAT3, were associated with high expression of NUPR1. The functional experiment was used to confirm the results of GSEA. E‐cadherin was significantly ameliorated in T24 and 5637 cells after NUPR1 interference. Conversely, the expression of Vimentin was significantly down‐regulated in the NUPR1 interference group compared with that in the control group. These results revealed that NUPR1 might exert its biological function in BLCA by affecting EMT. Moreover, we used the CIBERSORT algorithm to explore the association between NUPR1 and TICs in BLCA patients. We investigated the association between NUPR1 and 24 kinds of immune cells and revealed that macrophages, Th1 cells, neutrophils, and NK CD56 bright cells were the most related immune cells associated with the expression of NUPR1 in BLCA. We investigated the association between the NUPR1 expression and macrophages cell surface markers. The expression of NUPR1 was highly correlated with M0‐type macrophage markers, CD68, and ITGAM. There was a weak correlation between the expression of NUPR1 and the M1 macrophage marker, NOS2. No significant correlation was revealed between the expression of NUPR1 and the infiltration of M2 macrophages. The expression of NUPR1 was highly correlated with tumor‐associated macrophage markers including CCL2, CCR5, CD80, and CD86. Our results revealed that the expression level of NUPR1 may be involved in the polarization of macrophages to tumor‐associated macrophages. Moreover, the expression of NUPR1 gene was associated with the infiltration level of macrophages. NUPR1 is positively correlated with the M0‐type macrophage markers CD68 and CD11b‐integrin. Given that NUPR1 is up regulated in high‐grade BLCA, the combined detection of NUPR1 and macrophage markers CD68 and CD11b integrin may suggest that patients are more likely to develop high‐grade BLCA. There were some limitations in the present study. First, the molecular mechanism of NUPR1 involved in the polarization process of macrophages are warranted to be confirmed by further functional experiments. Second, in addition to cancer cells, there may be non‐cancer cells in the pathological sections. We invited professional pathologists to participate in the process of specimen selection, which can avoid the interference caused by non‐cancer tissues to the greatest extent. Third, the expression of GAPDH is not always stable. Although GAPDH was widely used in BLCA experiments,^46^, ^47^ adding another internal reference such as β‐actin can enhance the credibility of the current study. Additionally, the small compound ZZW‐115 was reported to compete with importins and lead to inhibition of NUPR1.^48^ Targeting the NUPR1 with the strong inhibitor ZZW‐115 is considered as a new strategy to treat pancreatic adenocarcinoma and hepatocellular carcinoma.^49^, ^50^ Using the inhibitor of NUPR1 to rescue the phenotype can better elucidate the mechanism of NUPR1 in BLCA. Moreover, it is helpful to further demonstrate the role of NUPR1 using another BLCA cell lines with lower NUPR1 expression than T24 or 5637 cell lines. Unfortunately, these functional experiments are warranted to be clarified by further studies in the future.

## 5 CONCLUSIONS

The expression of NUPR1 in BLCA tissues was significantly higher than in the control. The NUPR1 expression was significantly down‐regulated in T24 and 5637 cell lines after the interference of NUPR1. Moreover, in vivo experiment revealed that the tumor growth of BLCA can be delayed by inhibiting the expression of NUPR1. Signaling pathways, including EMT, apoptosis, and inflammatory were correlated with the expression of NUPR1. Additionally, in vitro experiments revealed that NUPR1 may exert its biological function in BLCA by affecting EMT. Furthermore, macrophages, Th1 cells, neutrophils, and NK CD56 bright cells were the most related immune cells associated with the expression of NUPR1 in BLCA. NUPR1 is positively correlated with the M0‐type macrophage markers CD68 and CD11b‐integrin. The current study reveals that NUPR1 serves as an oncogene and can be explored as a promising prognostic biomarker in BLCA.

## AUTHOR CONTRIBUTIONS

**Lifeng Zhang:** Conceptualization (equal); writing – original draft (lead). **Shenglin Gao:** Resources (equal). **Xiaokai Shi:** Formal analysis (equal); resources (equal). **Yin Chen:** Methodology (equal); resources (equal); validation (equal). **Shuzhang Wei:** Data curation (equal); software (equal). **Yuanyuan Mi:** Project administration (equal); writing – review and editing (equal). **Li Zuo:** Funding acquisition (equal); writing – review and editing (equal). **Chunjian Qi:** Conceptualization (equal).

## FUNDING INFORMATION

This study was supported by National Natural Science Foundation (No. 81902565), grant of Changzhou Health Commission Young Talent Project (No. CZQM2020065), Jiangsu Province 333 High‐level Talent Project. Changzhou health high‐level top talent (2022CZBJ057) and Changzhou No.2 People's Hospital Young Scientists Foundation (YJRC202039). Postdoctoral Research Project (BSH202214). Innovation team funding of Changzhou (XK201803).

## CONFLICT OF INTEREST

The researchers declared no competing interest.

## ETHICS APPROVAL

The current study has been approved by Ethics Committee of the Changzhou No.2 People's Hospital (Approval number: [2020]KY223‐01). All the patients have finished the written informed consent. Experiments containing human participants adhered to the Declaration of Helsinki. Experiments containing animals were based on the ARRIVE guidelines.

## Data Availability

The data generated in the current study are available by corresponding authors if the request is reasonable.
